# Comparison of different registration landmarks for MRI‐CT fusion in radiotherapy for lung cancer with post‐obstructive lobar collapse

**DOI:** 10.1002/acm2.12495

**Published:** 2018-11-22

**Authors:** Qian Han, Hengpo Liang, Peng Cheng, Dapeng Shi

**Affiliations:** ^1^ Department of Radiotherapy The People's Hospital of Zhengzhou University (Henan Provincial People's Hospital) Zhengzhou Henan China; ^2^ Department of Radiology The People's Hospital of Zhengzhou University (Henan Provincial People's Hospital) Zhengzhou Henan China

**Keywords:** lung cancer, mean displacement of the pulmonary artery images, MRI‐CT fusion

## Abstract

The registration of the two sets of images based on the spine and pulmonary artery landmarks and the geometric center difference of the mean displacement in the X, Y, and Z directions (X, Y, and Z represent the directions of the body from left to right, superior to inferior, and anterior to posterior) between their MRI‐CT fusions were compared, respectively. Fifty‐five lung cancer patients with post‐obstructive lobar collapse were enrolled in this study. Before radiation, two sets of simulating images according to the spine and the pulmonary artery registrations were obtained for each patient using MRI‐CT fusion. The differences of mean displacement in the X, Y, and Z directions based on spine and pulmonary artery landmarks were of −0.29, 0.25, and 0.18 cm, respectively. The mean displacements of the pulmonary artery based images in the three directions were smaller than that in the spine registration images (*P *< 0.05). By the method of pulmonary artery landmark, MRI‐CT has better registration accuracy and can better help confirm the target volume.

## 1 INTRODUCTION

Lung cancer is one of the most common causes of death among all cancers. Over the past 20 years, intensity‐modulated radiotherapy (IMRT) has rapidly developed as the main treatment method of lung cancer in radiation oncology. Accurate target volume delineation plays an important role in lung cancer radiotherapy. At present, the irradiation field setup and dose calculation are based on CT images, and the gross tumor volume (GTV) is contoured mostly from morphological features.^1^ However, in clinical practice, CT images are deficient in identifying the tumor volume, the extent of invasion, and metastatic lymph nodes, and there is some blindness in GTV delineation, especially for central lung cancer with post‐obstructive lobar collapse. Because of the decrease or disappearance of the alveolar gas content, which consolidates the CT image of the lung tissue and tumor fusion into a similar density of solid clumps, it is difficult to be distinguished from the tumor. PET‐CT improves the accuracy and specificity of diagnosis and the identification between atelectasis and tumor for the differential diagnosis of lymph node metastasis. In radiation oncology, PET‐CT is very helpful in target volume delineation, subsequently reducing the normal organ radiation dose while increasing the target dose and local control rate. However, because of its high cost, PET‐CT is not widely used in radiotherapy in our hospital. In recent years, magnetic resonance imaging (MRI) has been used for the diagnosis of lung cancer. MRI‐CT fusion plays an important role in head and neck tumor target delineation.^2^ However, there are some issues that need to be resolved. First, a stable and accurate automatic image registration and fusion method is the main issue. Second, the interpretation of the images is the ultimate goal of medical image fusion. Therefore, how to understand and use the image information should be explored. Third, there are many problems in image fusion; for example, there are uncertainties in the MRI‐CT fusion when patients suffer from advanced lung cancer accompanying post‐obstructive lobar collapse. We have accumulated some experience in the field of radiotherapy for dynamic contrast‐enhanced MRI‐CT fusion of esophageal cancer.^3^ Automatic registration is generally based on the spine. Because the esophagus and spine are closely adjoined, MRI‐CT registration is relatively easy. In lung cancer, it is difficult to reflect the real location of the tumor if MRI and CT imaging is registered based on the spine due to respiratory movements and heartbeat, and this may result in some errors. Image registration based on markers identified by the carina of the trachea has been reported in lung cancer.^4^ However, the carina is short and small and is difficult to use for MRI‐CT registration of lung cancer.

Lung cancer, which causes post‐obstructive lobar collapse, is often located in the hilum. The pulmonary artery volume and its span near the bilateral hilum pulmonis are large. This paper evaluates the pulmonary artery as a MRI‐CT landmark to identify lung tumors.

## 2 MATERIALS AND METHODS

### 2.A Patients

A total of 55 lung cancer patients with post‐obstructive lobar collapse were selected from March 2014 to November 2016. The patients’ clinical characteristics are listed in Table 1. All the patients’ pathological results were confirmed by our hospital. They had complete imaging data, and imaging diagnosis was lung cancer with atelectasis, and the stages were III–IV. The patients’ status scores were KPS ≥ 70.

**Table 1:** Patient characteristics

	*n*	Ratio (%)
Sex		
Male	49	89.1
Female	6	10.9
Age (yr)		
Median (range)	58 (43–80)	
≤60	32	58.2
>60	23	41.8
Tumor histology		
SCLC	28	50.9
Squamous carcinoma	25	45.5
Adenocarcinoma	2	3.6
Stage		
IIIA	16	29.1
IIIB	20	36.4
IV	19	34.5
Location of primary tumor		
Left upper lobe	12	21.8
Left lower lobe	9	16.4
Right upper lobe	18	32.7
Right middle lobe	6	10.9
Right lower lobe	10	18.2

A written informed consent was obtained from all patients and the protocol was approved by the ethics committee of The People's Hospital of Zhengzhou University.

### 2.B Instruments

The Instruments used in this study included: Treatment planning system (TPS Pinnacle 9.8), Sixteen‐slice CT simulator (GE Discovery CT590 RT), Laser positioning system (LAP), and MRI (GE Discovery MR750).

### 2.C Study methods

#### 2.C.1 CT positioning

Patients were scanned on a CT simulator under calm breathing. Most patients were simulated with intravenous contrast (1.0 ml/kg, 320 mg/100 ml iodine). All patients were in the supine position and immobilized in a thermoplastic body mold. The CT scan images were transferred to a treatment planning system.

#### 2.C.2 Image fusion

Pretreatment MRI images were acquired from all patients to delineate the GTV. The MRI images were fused to the CT planning images.

Fusion MRI‐CT imaging for contouring spine and pulmonary arteries was performed.

Pulmonary arteries, spine, and lesions (including tumors and tumors secondary to atelectasis) were delineated on the CT and MRI image. MRI and CT were fused according to pulmonary artery and spine. However, the size of the same lesion observed by fused image was not exactly the same on CT and MRI. Then, we manually adjusted the fused image according to the contour range of CT and MRI to meet the fusion quality. The spine and the pulmonary artery registry‐based MRI‐CT fusion were obtained according to the lesion range of the patients, respectively. The geometric center coordinates are automatically calculated by the treatment planning system. The difference in the geometric center described here refers to the magnitude of the change in the geometric center coordinates after manual adjustment of the two fusion images (spine and pulmonary artery registration). Finally, the difference of the coordinate displacement data before and after the manual adjustment is compared, and the conclusion is drawn. Two sets of MRI‐CT fusion (spine and pulmonary artery registrations) were obtained from 55 patients.

#### 2.C.3 Imaging evaluation

**Figure 1:**
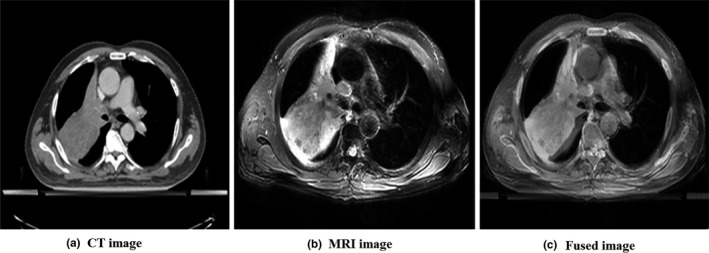
Image registration based on the spine. (a) CT image, (b) MRI image, and (c) fused image.

**Figure 2:**
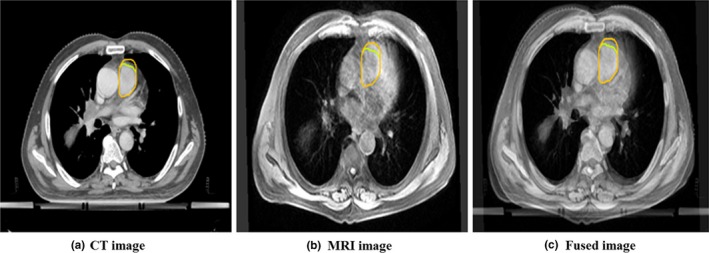
Image registration based on the pulmonary artery. (a) CT image, (b) MRI image, and (c) fused image.

The different geometric centers between spine and pulmonary artery registrations were compared by the mean displacement (MD) of the atelectasis area in the X, Y, and Z directions (X, Y, and Z represent the directions of left to right, superior to inferior, and anterior to posterior, respectively. Figures 1 and 2). In order to ensure the accuracy and repeatability of the coordinate delineation, all the delineations were completed by a deputy chief physician. The other two deputy chief physicians reviewed and discussed them and reached an agreement. The pulmonary arteries delineate the lower edge of the aortic arch to the lower edge of the right pulmonary artery and the right pulmonary vein.

## 3 STATISTICAL ANALYSES

A database was created and statistics were calculated using SPSS 13.0 (SPSS, Inc., Chicago, IL, USA). The data between the two groups were analyzed using the rank sum test. The errors were calculated using the landmark registration method. According to the standard α = 0.05, *P* < 0.05 was statistically significant.

## 4 RESULTS

The differences of MD in the X, Y, and Z directions between the two MRI‐CT fusions (according to spine and pulmonary artery registrations, respectively) were −0.29, 0.25, and 0.18 cm, respectively. The MD of the pulmonary artery image was less than that of the spine image (*P *< 0.05, Tables 2 and 3). According to the Response Evaluation Criteria in Solid Tumour (RECIST version 1.1), the short‐term clinical efficacy 4 months after radiotherapy is shown in Table 4. According to the RTOG/EORTC classification criteria for early and late radiation reactions, none of the studied patients had gastrointestinal adverse effects, radiation pneumonitis, and bone marrow arrest greater than grade 2.

**Table 2:** The mean displacement of the X, Y, and Z directions

	Average (cm)	Standard deviation	Mean standard error
X spine^a^	−1.55	0.89	0.11992
X lung	−1.26	1.02	0.13713
Y spine	3.38	1.59	0.21461
Y lung	3.13	1.58	0.21327
Z spine	1.73	1.38	0.18631
Z lung	1.55	1.38	0.18663

a −1.55 cm here indicates that it moves more to the left than −1.26 cm.

**Table 3:** The differences in the X, Y, and Z directions of the two groups

	Mean ± SD	Paired difference		
		*P* value	95% confidence interval	
			Lower limit	Upper limit
X spine‐X lung	−0.29 ± 0.07	= 0.000	−0.42405	−0.14202
Y spine‐Y lung	0.25 ± 0.08	= 0.002	0.09796	0.41113
Z spine‐Z lung	0.18 ± 0.06	= 0.008	0.04661	0.30302

**Table 4:** Short‐term clinical efficacy

	CR	PR	SD	PD	RR (CR + PR)
Patients	0	23	26	6	23
Ratio (%)	0	41.8	47.3	10.9	41.8

CR, complete response; PR, partial response; SD, stable disease, PD, progressive disease; RR, response rate.

## 5 DISCUSSIONS

Medical image fusion is widely used in medical science.^5^, ^6^ Its advantage is that it can synthesize the multi‐modal imaging information and be helpful in diagnosis and treatment. However, the dose calculation is based on tissue density; the target (cancer) is often difficult to be distinguished from the objects that have similar densities even in enhanced CT scanning. This makes lung radiotherapy very difficult.

For more precise positioning of tumors, studies of image registration methods have been reported, such as the choice of registration points, gray similarity of the organization's registration, and so on.^7^ Image registration is the basis of fusion, and its accuracy directly affects the accurate location and size of the target volume.^4^, ^8^

In this study, we considered the effect of respiratory movement and heartbeat on the location of lung tumors. We noted that it was difficult for the conventional positioning device to be used for MRI scanning. Because the patients were not in the same position during the CT and MRI scans, and the traditional spine registration image fusion could cause large errors. Hence, we used the pulmonary artery as another registration marker. The MRI and CT images of the same patient were fused with different registration anatomical markers. The differences between these two groups were compared and the group with smaller differences in the combination can be considered a more reliable registration marker. In the MRI‐CT fusion based on the spine, the mean values of displacement were −1.55, 3.38, and 1.73 cm in the X, Y, and Z directions, respectively. In the MRI‐CT fusion based on the pulmonary artery, the mean values of displacement were −1.26, 3.13, and 1.55 cm, respectively. The displacement of the spine as the registration marker is higher than that of the pulmonary artery (where the negative value represents the direction, and the displacement is the absolute value). The differences in the X spine‐X lung, Y spine‐Y lung, and Z spine‐Z lung were 0.29 cm (*P* = 0.000), 0.25 cm (*P* = 0.002), and 0.18 cm (*P* = 0.008), respectively. The displacement of the pulmonary artery as a registration landmark was significantly lower than that of the spine.

Lung cancer, which causes atelectasis, is mainly central lung cancer, which is anatomically closer to the pulmonary artery. Based on our previous image registration experience in radiotherapy for esophageal cancer,^3^ the esophageal stent is better than the spine as a registration landmark, which reduces the effects of breathing and heartbeat factors on chest tumor imaging.

In this study, we also conducted an observation on the efficacy of 52 patients who underwent multi‐cycle chemotherapy (>4 cycles) and three patients who completed radiotherapy within two cycles. It was difficult to define sequential or concurrent radiotherapy and chemotherapy. In most cases, radiotherapy was used after chemotherapy failure. This study investigated the effectiveness of local radiotherapy with an objective response rate (CR + PR) of 41.8%. The objective response rate of randomized radiotherapy reported in the literature is 43%.^9^, ^10^, ^11^, ^12^, ^13^ During the follow‐up, these patients had no more than grade two radiation pneumonia, gastrointestinal reactions, and bone marrow suppression. This study showed that the target volume determined by pulmonary artery registry based MRI‐CT can improve the reliable short‐term efficacy.

## 6 LIMITATION OF THIS STUDY

MRI is not a preferred modality for delineation of target volume in lung cancers since the distortion associated with MRI is quite high. Hence, an alternate MRI protocol shall be considered for the further study. As well, MRI should be used in combination with CT. It has also been reported that diffusion‐weighted MRI has the same effect as PET‐CT.^14^, ^15^, ^16^, ^17^ Respiratory movements can also result in a poor quality of chest MRI‐CT fusion. Although patients in this study used calm breathing because theoretically the pulmonary arteries and lesions are close in position, the results of the actual studies still show the presence of image center displacement. The same respiratory phase MRI‐CT fusion will be explored in the future. In addition, we should pay attention to new MRI technology, such as functional MRI, which is an appropriate fusion method for lung cancer target sketches. In the future, the fusion of functional and anatomical images should be considered.

## 7 CONCLUSIONS

The MD of the geometric center in X, Y, and Z directions in the pulmonary artery based image was less than that in the spine fusion. Pulmonary artery MRI‐CT fusion can help confirm target volumes.

## ETHICS APPROVAL

A written informed consent was obtained from all patients and the study protocol was approved by the ethics committee of The People's Hospital of Zhengzhou University(Henan Provincial People's Hospital) Hospital.

## CONFLICT OF INTEREST

The authors declare that they have no competing interests.
